# Drug-free tracheal intubation by specialist paramedics (critical care) in a United Kingdom ambulance service: a service evaluation

**DOI:** 10.1186/s12873-021-00533-0

**Published:** 2021-11-20

**Authors:** Silas Houghton Budd, Eleanor Alexander-Elborough, Richard Brandon, Chris Fudge, Scott Hardy, Laura Hopkins, Ben Paul, Sloane Philips, Sarah Thatcher, Paul Winsor

**Affiliations:** Critical Care Operating Unit, South East Coast Ambulance Service NHS Foundation Trust, Nexus House, 4 Gatwick Road, Crawley, RH10 9BG UK

**Keywords:** Tracheal intubation, Intubation, Airway, Paramedic, Prehospital, Critical care

## Abstract

**Background:**

Drug-free tracheal intubation has been a common intervention in the context of out-of-hospital cardiac arrest for many years, however its use by paramedics has recently been the subject of much debate. Recent international guidance has recommended that only those achieving high tracheal intubation success should continue to use it.

**Methods:**

We conducted a retrospective service evaluation of all drug-free tracheal intubation attempts by specialist paramedics (critical care) from South East Coast Ambulance Service NHS Foundation Trust between 1st January and 31st December 2019. Our primary outcome was first-pass success rate, and secondary outcomes were success within two attempts, overall success, Cormack-Lehane grade of view, and use of bougie.

**Results:**

There were 663 drug-free tracheal intubations and following screening, 605 were reviewed. There was a first-pass success rate of 81.5%, success within two attempts of 96.7%, and an overall success rate of 98.35%. There were ten unsuccessful attempts (1.65%). Bougie use was documented in 83.4% on the first attempt, 93.5% on the second attempt and 100% on the third attempt,

**Conclusion:**

Specialist paramedics (critical care) are able to deliver drug-free tracheal intubation with good first-pass success and high overall success and are therefore both safe and competent at this intervention.

## Introduction

### Background

Tracheal intubation (TI) is a common intervention in critical care medicine and is indicated for a number of reasons, including airway protection in comatose patients or those at risk of aspiration, facilitation of mechanical ventilation, and in anticipation of a deteriorating clinical course that will very likely lead to respiratory failure [15]. In prehospital emergency medicine, TI is used in the context of both prehospital emergency anaesthesia or without anaesthetic drugs in the context of out-of-hospital cardiac arrest (OHCA) – drug-free tracheal intubation (DF-TI). While paramedic-led anaesthesia is delivered safely and effectively abroad [8], in the United Kingdom (UK) it is currently restricted to physician-led services, such as air ambulance charities [1] [16].

Drug-free tracheal intubation in the context of OHCA has been a core intervention for paramedics in the UK for over 30 years and is indicated when less invasive methods of airway care (such as supraglottic airway devices) are deemed suboptimal, although there is ongoing debate about both its safety and efficacy [21]. In 2015, the European Resuscitation Council [28] recommended TI as the most reliable method of securing the airway in OHCA, however also highlighted that there were no reliable data showing increased survival with this method and went on to recommend the use of a supraglottic airway device as an acceptable alternative. Given the lack of survival benefit associated with TI and the efficacy of supraglottic airway devices, the most recent consensus from the International Liaison Committee on Resuscitation recommended a supraglottic airway device for OHCA in settings with “low TI success” and supraglottic airway device or TI for OHCA in settings with “high TI success” but did not define “high” or “low” [27]. The most recent European Resuscitation Council and Resuscitation Council UK guidelines [25, 26] recommend that those with a high TI success rate should use TI and define this as greater than 95% within two attempts. Given supraglottic airway devices are now so widely available, a recent consensus statement from the College of Paramedics [4, 5] in the UK recommended that only paramedics with ongoing education, training, and clinical governance should perform TI, a recommendation which is emphasised by Soar et al. [26].

Tracheal intubation can be a complex procedure and may lead to unrecognised oesophageal intubation, long pauses in closed chest compressions, prolonged durations of hypoxia, or other complications if not achieved quickly [2]. Evidence suggests that multiple attempts at TI are associated with an increased risk of adverse events [11] and as such, first-pass success – successful placement of the tracheal tube in the trachea following the first attempt at laryngoscopy and confirmed by waveform capnography – is a recognised standard against which TI success is measured [24]. In the recent, large ‘PART’ trial (*n* = 3004) conducted in the United States of America, first-pass success in paramedic DF-TI was just 52% [30] and second-pass success was not reported. Meanwhile, the concomitant ‘AIRWAYS-2’ trial in the UK (*n* = 9296) found success after up to two attempts to be 79% (and did not report first-pass success) [2].

Most paramedics in the UK attend an average of three OHCA per year [23] and as such, exposure to TI is likely to be low. However, the advent of specialties in paramedic practice [4, 5] has led to the development of specialist paramedics in critical care (SPCC) who are specifically tasked to high acuity cases, including airway complications and OHCA. SPCCs in South East Coast Ambulance Service NHS Foundation Trust (SECAmb) have a minimum of 3 years’ post-registration experience as a paramedic prior to a two-year postgraduate diploma in prehospital critical care in which they receive additional training and education on airway care, as well as six weeks of clinical placement in intensive care units and theatres where this learning is consolidated in practice. SPCCs also undertake regular simulation training in airway care and engage in structured clinical governance sessions where complex cases are discussed in detail.

The large international trials mentioned do not differentiate between specialist and non-specialist paramedics despite the differences in experience, education, and exposure which may affect DF-TI success. For instance, the most recent data from SPCCs in London Ambulance Service showed first-pass success of 90.8% and overall success of 96.4% [17]. These data suggest SPCCs may have higher DF-TI success than non-specialist paramedics however are only from one service in one geographical region. To further address this knowledge gap, we aimed to conduct a similar service evaluation of SPCCs in SECAmb.

## Methods

### Aim

We aimed to assess DF-TI success when performed by SECAmb SPCCs by conducting a retrospective database review.

### Study design

This was a retrospective service evaluation using routinely collected data from the SECAmb clinical governance database – CCPBase [18]. This is a secure, online reporting system which stores anonymised, clinical reports for the benefit of audit, reflection, and learning.

### Settings

SECAmb serves a geographical area of approximately 3600 mile^2^ with a resident population of 4.5 million and a transient population of eight million [7]. Data were retrieved from CCPBase for the period between 1st January – 31st December 2019.

### Participants

All patients receiving DF-TI by SPCCs were identified for analysis. Records with incomplete data or where the DF-TI was completed by a non-SPCC were excluded. As discussed, all CCPBase records are anonymised and only the relevant variables were exported for analysis.

### Outcomes

The primary outcome was first-pass success, and secondary outcomes were success within two attempts, overall success, grade of laryngoscopy view, and use of bougie.

The Cormack-Lehane classification system (CLCS) is the model used by SPCCs in SECAmb to document and communicate the anatomical view achieved by the rescuer during direct laryngoscopy. It classifies the view achieved with direct laryngoscopy from easy to difficult using the numbers 1–4 [6]. In addition to these four views, CCPBase has two further dropdown options: ‘impossible’ for when anatomy or injury precludes laryngoscopy and ‘unrecognisable’ for when a CLCS grade of view is not achieved.

The use of a bougie to optimise TI success has been widely accepted in prehospital care for a number of years and is recommended in SECAmb for this reason.

### Data sources / measurement

CCPBase has a built-in proforma for documenting TI. Dropdown boxes are used to record the number of attempts, as well as other factors such as the grade of view achieved, size of bougie used, or the presence of airway soiling. During the period of data collection there was a disparity in the way in which SPCCs recorded DF-TI attempts. Some documented the number of attempts by SPCC only while others documented the number of attempts the patient received (including non-SPCC and SPCC). In addition to the dropdown boxes however there was a free text box alongside the proforma, in which SPCCs could document further details about the intervention, such as the person delivering each attempt at DF-TI. This was reviewed for further information about the person delivering the intervention to ensure accuracy of data.

### Bias

A potential bias was present as all the authors are employed as SPCCs by SECAmb and thus had a vested interest in achieving positive results [29]. This may have led to a conscious or subconscious bias in the free text box analysis described previously as this may have been open to interpretation [13]. To account for this, cases where it was unclear who performed the intervention were excluded from analysis.

### Statistical methods

Descriptive statistical analysis was undertaken to report means, medians, and percentages.

## Results

### Participants

**Fig. 1:**
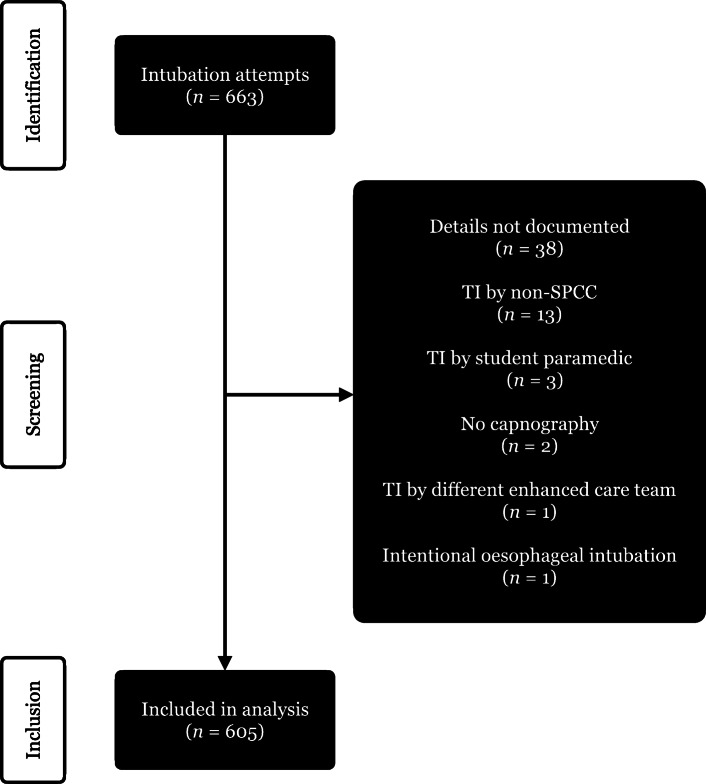
Data flow

A data flow diagram is available in Fig. 1. There were 663 DF-TI records recorded on CCPBase between 1st January – 31st December 2019. Of these, 513 (84.8%) reported the use of a bougie, while the remainder had no reported bougie use. Fifty-eight records were excluded, 38 because the details were not documented, 13 in which TI was performed by non-SPCC, three in which TI was performed by student paramedics, one which was completed by another enhanced care team, and one which was intentionally placed in the oesophagus prior to DF-TI. In two records, the tracheal tube was documented as visibly in the trachea but without waveform capnography and the capnography device was subsequently identified as not having been functioning. These were excluded as although a successful intubation, they did not meet our definition for a successful TI which included a waveform capnogram. One of these was a grade 1 view while the other was a grade 2 so the results are unlikely to have affected our final analysis significantly. Following exclusion, there were 605 remaining records which were fully analysed.

### Descriptive data

Median (range) age was 63 (0–98), mean (range) estimated weight was 84.7 kg (3–222), and there was a higher proportion of male patients than female (417; 69%). There were 15 paediatric patients (0.2%), of which seven were below one year of age and three were between zero and five years of age.

### Main results

The main results are available in Tables 1 and 2. TI success rate was 98.35% (*n* = 595) overall. Of the ten unsuccessful TI attempts, none had documented failed ventilation. Four hundred and ninety-three were intubated on the first attempt (81.5%), 92 on the second attempt (15.2%), and 10 on the third attempt (1.6%). Bougie use was documented in 411 on the first attempt (83.4%), 86 on the second attempt (93.5%) and 10 on the third attempt (100%), Cormack and Lehane scores were grade 1: 50.6% (*n* = 306), grade 2: 29.9% (*n* = 181), grade 3: 14.4% (*n* = 87), and grade 4: 3.1% (*n* = 19). There were three ‘impossible’ view (0.5%) and eight ‘unrecognisable’ views (1.3%) recorded. The remaining record had no information on grade of view.

**Table 1:** Tracheal intubation success

Attempts	***n***	%	Combined (%)	Bougie (%)^a^
1	493	81.5		83.4
2	92	15.2	96.7	93.5
3	10	1.65	98.35	100
Not successful	10	1.65	–	

^a^Cases in which bougie use was documented

**Table 2:** grade of view

Cormack-Lehane grade	***n***	%
1	306	50.6
2	181	29.9
3	87	14.4
4	19	3.1
Impossible	3	0.5
Unrecognisable	8	1.3
Not recorded	1	0.2

## Discussion

### Key results

We found a first-pass success rate of 81.5% which is lower than that of other SPCCs (90.8%) [17] but comparable with that of prehospital physicians (84.4, 84.8%) [12] [14]. While there is no universally accepted rate of first-pass success for TI, a recent systematic review and meta-analysis incorporating > 40,000 emergency department TIs in 83 institutions worldwide found the average first-pass success to be 84.1% [22]. Compared with TI in the emergency department, DF-TI in the context of OHCA is often performed on the ground, and complicated by factors such as an unpredictable environment, poor lighting, or an unfamiliar team – all of which may make the procedure more complicated. Although Park et al. [22] found no statistically significant association between the proportion of TI performed during cardiac arrest and first-pass success, a recent study of TI success by prehospital anaesthetists (*n* = 1006) found a significantly lower rate of first-pass success in those receiving cardiopulmonary resuscitation (CPR) versus non-CPR (84.4% vs 91.4%, *p* = 0.01) [12], suggesting the procedure may be more difficult in this context.

The most recent European Resuscitation Council and Resuscitation Council UK guidelines [25, 26] echo the recommendation by the International Liaison Committee on Resuscitation that only those working within a system that can demonstrate high TI success should use this technique [27] and define “high TI success” as greater than 95% within two attempts (expert consensus). We found success within two attempts to be 96.7%, which is again comparable with other enhanced care teams. SPCCs from London Ambulance Service demonstrated success within two attempts for DF-TI in OHCA as 96.4% [17] while prehospital physicians from London’s Air Ambulance reported 98.8% (89.6% of records during prehospital emergency anaesthesia, 10.4% during OHCA). On the other hand, non-specialist paramedics in the ‘AIRWAYS-2’ trial achieved success within two attempts just 79% of the time [2].

Our analysis demonstrated a difference between successful TI on the first attempt and that within two attempts (81.5% vs 96.7%, respectively). There are several reasons why this may be, not least the complexity of delivering successful TI in an undifferentiated patient within an inherently suboptimal environment. This difference could also be exacerbated by incomplete or suboptimal preparation of the environment, patient, equipment and/or team members involved with the intervention. Although we found successful TI within two attempts to be 96.7%, and overall success of 98.35% (after three attempts), ensuring full and robust preparation would likely serve to optimise the very best first-pass success achievable, in turn mitigating the opportunity for the patient to suffer deleterious consequences of the care provided.

Although it has been shown that there can be significant inter-operator variability when assessing the laryngoscopy view using the CLCS [20] this detail was included in order to consider any obvious trends. In this analysis 487 (80.5%) of the records reported a CLCS grade 1 or 2 suggesting less likelihood of complication encountered during TI. Thirty (4.9%) of all records were described as grade 4 view, unrecognisable, or impossible inevitably resulting in extremely challenging or unsuccessful TI. Analysing the reason for unrecognisable or impossible views was outside the scope of this evaluation.

The use of a bougie to optimise TI success has been widely accepted in prehospital care for a number of years, following a pivotal paper by Driver et al. [9]. A recent secondary analysis of the ‘PART’ trial data [3] found bougie use for TI during OHCA to increase first-pass success (52.1% vs 43.8%), although this was not significant after adjusting for confounders (OR 1.12, 95% CI: 0.97–1.39). There is a paucity of evidence supporting the use of a bougie by SPCCs for DF-TI during OHCA, however given the evidence discussed it is the most routinely used TI adjunct in SECAmb (as opposed to a malleable stylet). We found bougie use to be documented in 84.8% of cases which is far higher than by paramedics in the ‘PART’ trial (35.9%) [3]. CCPBase was not optimised to record the reason for not using a bougie and so the remaining 15.2% may represent records in which either no adjunct or a malleable stylet was used, or in which a bougie was used but not documented.

During data collection we noted a disparity in the way SPCCs record their TI attempts. Some documented the number of attempts by SPCC only while others documented the number of attempts the patient received (including non-SPCC and SPCC). We addressed this by analysing the associated free text, however it may have led to some inaccurate data. Additionally, 38 of the records that were excluded during screening were because the TI data had not been reported and this may have had an impact on our results.

Finally, the data were all self-reported and therefore subject to both recall and reporting bias. As this was a retrospective database review however the SPCCs were not aware of the study design at the time of recording the TI so these biases are likely to be limited.

### Interpretation

Our findings suggest that SECAmb SPCCs can facilitate DF-TI at a standard comparable to that of other enhanced care teams within prehospital care. An overall success rate of 98.35% is comparable with prehospital emergency anaesthesia data from both physicians (99.8%) [10] and SPCCs abroad (99.4%) [8] and is similar to the rate of overall success for the only other UK SPCC data that the authors are aware of (96.4%) [17].

We demonstrated a lower first-pass success rate than similar services, the reasons for which have been discussed. Our high overall success rate and success within two attempts however meets the definition of “high TI success” recommended by the European Resuscitation Council and Resuscitation Council UK [25, 26] and demonstrates SECAmb SPCCs are both safe and competent at DF-TI.

### Generalisability

Specialist paramedics (critical care) in SECAmb have a different level of education and experience to non-specialist paramedics, but similar to that of SPCCs in other services. External validity is therefore limited in terms of the paramedic profession, but good in relation to other SPCC teams in the UK and abroad.

## Conclusion

We have demonstrated that SECAmb SPCCs achieved good TI success with high overall success and acceptable first-pass success across the study period. These data show that appropriately trained paramedics can perform DF-TI safely and effectively.

## Data Availability

The datasets used and/or analysed during the current study have not been made publicly available but have been retained for 1 year (until 30/04/2022) and are available from the corresponding author on reasonable request.

## Abbreviations

CLCS: Cormack-Lehane Classification System
CPR: Cardiopulmonary Resuscitation
DF-TI: Drug-free Tracheal Intubation
OHCA: Out-of-hospital Cardiac Arrest
OR: Odds Ratio
SECAmb: South East Coast Ambulance Service
SPCC: Specialist Paramedic (Critical Care)
TI: Tracheal Intubation
UK: United Kingdom
